# Research progress on the intervention of cognitive function using transcranial alternating current stimulation technology

**DOI:** 10.3389/fpsyg.2024.1405636

**Published:** 2024-11-15

**Authors:** Qingchang Wu, Changli Wu, Jian Liu

**Affiliations:** ^1^College of Physical Education, Soochow University, Suzhou, Jiangsu, China; ^2^Hubei International Travel Health Care Center (Outpatient Department of Wuhan Customs Port), Wuhan, China; ^3^Physical Education School of Shenzhen University, Shenzhen, Guangdong, China

**Keywords:** transcranial alternating current stimulation, cognitive function, intervention effects, application strategies, ethical

## Abstract

Transcranial Alternating Current Stimulation (tACS) is a non-invasive brain stimulation that stimulates the cerebral cortex through the output current to regulate neural excitability. This review systematically summarizes the research results of tACS on working memory, learning ability, and decision-making ability, and analyzes the application schemes, safety, and unresolved issues of tACS in the field of cognitive function to provide a theoretical reference for the application of tACS in the field of cognition. Research has found that: (1) tACS intervention can improve the working memory, learning ability, and exercise decision-making ability of athletes and healthy individuals and has a positive effect on improving exercise performance. (2) The factors that determine the effectiveness of tACS intervention include stimulation frequency, stimulation phase, stimulation area, and stimulation dose. The stimulation area and frequency determine which cognitive function tACS affects, whereas the stimulation phase and dose determine the magnitude of the intervention effect. Moreover, before practical application, individual cognitive status, age level, and timing of application should be included in the factors that affect the effectiveness of tACS intervention to develop more scientific intervention plans. (3) Despite the absence of evidence indicating significant safety issues associated with the use of tACS, its widespread adoption among athletes still poses safety risks under the World Anti-Doping Code. In competitive sports, whether the use of tACS will be classified as a “neuro-doping” method leading to disqualification remains uncertain. Therefore, authoritative institutions to provide comprehensive guidelines on the application of tACS, clearly delineating its usage scenarios and defining the safety parameters for tACS stimulation. Additionally, the development of detection devices for tACS usage is essential to ensure that any intervention using tACS can be monitored effectively.

## 1 Introduction

The brain is the foundation of human movement and cognition, playing a crucial role in improving motor performance and cognitive function (Wu et al., 2021). In recent years, the relationship between sports performance and cognitive function has received much attention, and advances in neuroscience have made it possible to explore the relationship between motor performance and cognitive function (Yin et al., 2014). The improvement of cognitive function often promotes good motor performance, which has been confirmed by many studies (Hockey, 1993; Cao, 2016; Zhao et al., 2021; Tod et al., 2015; Kamali et al., 2019). At present, brain neural regulation technology is an important means to improve cognitive function in the brain. Transcranial Electrical Stimulation (TES) is a non-invasive brain neural regulation technology that activates cortical neurons to improve cognitive function by stimulating electrodes to apply low-intensity currents to specific brain regions. TES can be divided into transcranial direct current stimulation (tDCS) and transcranial alternating current stimulation (tACS) based on different current forms. Among them, tDCS has achieved good results in the fields of exercise and rehabilitation, improving the exercise performance of users. Unlike tDCS, tACS can induce more persistent synaptic changes through frequency oscillations and peak time plasticity, resulting in a longer duration of action of tACS (Kasten and Herrmann, 2017). And because the stimulation of tACS is bipolar current, the side effects of tACS are smaller, which has attracted the interest of many researchers (Antal et al., 2017).

Cognitive function is a complex brain activity encompassing working memory, learning ability, motor decision-making, and visuospatial skills (Ren et al., 2013; Fresnoza et al., 2020; Hwang et al., 2022). In actual sports activities, not only is physical participation required, but cognitive involvement is also necessary. The more complex the sports scenario, the deeper the cognitive involvement needed, often requiring more cognitive resources to handle changes in the sports situation, such as analyzing action techniques and sports scenarios, and making decisions under pressure. These factors collectively increase the cognitive load on athletes, including working memory and motor decision-making (Borson, 2010). Prolonged cognitive engagement can lead to cognitive fatigue, where excessive energy consumption by the brain results in a temporary decline in cognitive performance. This decline can adversely affect sports performance, reduce competition results, and even cause sports injuries (Holtzer et al., 2011; Van Cutsem et al., 2017). Research has found that working memory, learning ability, and sports decision-making are closely related to sports performance, with almost every type of sport involving these three cognitions (Tang et al., 2020; Zhu et al., 2015). Furley et al. found in their research that the improvement of sports performance depends on working memory, decision-making, skill acquisition, sensory perception, and stress (Furley et al., 2010). Working memory is the foundation of other higher-lever cognitive functions, and almost all cognitive functions are based on working memory, such as motor learning and motor decision-making. In the process of mastering motor skills, the stronger the working memory ability and motor learning ability, the faster the speed of mastering sport skills, the higher the degree of sport automation, and the better the sport performance (Zhou et al., 2018; Kal et al., 2016). For sports competitions with complex sports scenarios, higher-level motor decision-making is needed to improve their performance. Excellent athletes are usually able to make quick decisions to adapt to changes in the competition more quickly. Accurate decision-making can reduce the chances of errors, help athletes effectively execute tactics and strategies, and also effectively leverage their technical and physical advantages to improve their sports performance and improve competition results (Amann and Secher, 2010; Huijgen et al., 2015; Head et al., 2017). Moreover, these three cognitive functions have certain similarities in the functional areas of the brain, involving most areas of the brain, and the main functional areas are in the frontal and parietal lobes (Smith and Jonides, 1999; Meissner et al., 2018; Gaudry and Kristan, 2012). tACS has increasingly been used as a neuroregulatory technique to modulate cognitive abilities, even becoming a crucial means to enhance cognition (Tavakoli and Yun, 2017). However, the current application effects of tACS in the cognitive domain are mixed, necessitating scientific application paradigms to guide the use of tACS in the cognitive field to improve sports performance.

Based on the above reality, this study summarizes the relevant research on tACS intervention on working memory, motor learning, and motor decision-making in the past 30 years, analyzes the effect of tACS on the above cognitive functions, summarizes the practical application plans and safety issues of tACS, and provides theoretical basis and application guidance for the application of tACS in the cognitive field of athletes, to help athletes, coaches, and researchers better utilize tACS to participate in sports training and competitions.

## 2 Overview of transcranial alternating current stimulation

### 2.1 Definition and parameters

tACS stimulates the cerebral cortex by outputting sinusoidal alternating currents of different frequencies, where the voltage gradually changes from positive to negative every half cycle. Therefore, the current flows from the anode electrode to the cathode electrode within one and a half cycles, and in the second half cycle, it flows in the opposite direction (Ruffini et al., 2013). Clinically, different frequencies of current are commonly used to regulate brain oscillations and induce brain function (Antal and Paulus, 2013). At present, the common stimulation frequencies of tACS include 6.5, 10, 20, 40, 50, and 70 Hz, etc. The duration of current stimulation is mostly between 20 and 30 min, and the area of the stimulation electrode is 16, 25, and 35 cm^2^. The peak-to-peak intensity of current stimulation is generally between 0.5 mA and 2 MA (Klink et al., 2020).

### 2.2 Physiological mechanisms

tACS stimulates the cerebral cortex through sinusoidal alternating current, and its mechanism of action can be divided into the following: exogenous oscillations induce endogenous oscillations in the brain; Inducing synaptic plasticity to regulate brain function; tACS regulates endogenous brain oscillations in a frequency-specific manner by applying a specific frequency of current to the cerebral cortex, altering the membrane potential of dendrites or axons in an oscillatory manner. This oscillation can interact with natural oscillations in the distant cerebral cortex, ultimately triggering the excitation of brain neurons. tACS can emit current frequencies related to cognitive function, thereby affecting cognitive ability Applying tACS stimulation slightly higher or lower than the intrinsic frequency of brain regions can accelerate or decelerate intrinsic oscillations (Vöröslakos et al., 2018; Raco et al., 2014).

## 3 Research progress on improving cognitive function through transcranial alternating current stimulation

In recent years, tACS has received more attention due to its AC characteristics and safety. Some research on tACS has mostly focused on promoting motor skills and cognitive regulation (Zhang and Lv, 2024; Li et al., 2023; Zhang and Li, 2022). Scholars Qian et al. pointed out that cognitive functions mainly include attention, working memory, executive power, decision-making power, and multitasking ability (Qian et al., 2020). Klink et al.'s research found that working memory, motor learning, and decision-making power are highly correlated with motor performance (Klink et al., 2020), which is one of the prerequisites affecting motor performance. The complexity of cognitive function is high, and individual differences are significant (Shaw et al., 2020; Lövdén et al., 2020). Therefore, when using tACS to intervene in cognitive function, the results often have differences. This review reveals the effects of tACS on different cognitive functions, summarizes the application plans of transcranial alternating current, and provides a reference for the application of tACS in the cognitive field.

### 3.1 Impact of transcranial alternating current on working memory

This study found that tACS can improve working memory in healthy individuals (Bender et al., 2019). However, the selection of frequency has specificity. Compared with a frequency of 7 Hz, a frequency of 4 Hz can better improve working memory levels. The reason is that the effect of tACS on working memory propagates along the brain network. When the induced frequency emitted by tACS matches the endogenous rhythm, the entrainment effect is better. And theta oscillation acts as a gating mechanism in working memory, providing optimal neural conditions for specific processing (Jaušovec et al., 2014) found that the oscillation frequency of 4 Hz is closer to that of the human brain theta frequency, therefore, a 4 Hz frequency has a significant impact on an individual's working memory. Scholar Borghini et al. analyzed from the perspective of θ-γ phase coupling theory and believed that slower theta waves (4 Hz) allow more gamma cycles to be nested within slower theta waves (4 Hz) compared to faster theta waves (7 Hz), thereby improving working memory capacity (Borghini et al., 2018). Scholar Sauseng et al. also supports this viewpoint and believes that multiple gamma wavebands can be nested within theta frequency band, it can promote instantaneous memory of multiple items, ultimately increasing working memory capacity (Sauseng et al., 2019). Roux and Santarnecchi found in their study that when cognitive load capacity is high, the stimulation effect of gamma band tACS is more significant. Although γ- tACS has a positive impact on improving working memory, the improvement effect also varies. When cognitive load is higher, tACS prioritizes improving performance in more complex cognitive tasks. Santarnecchi and colleagues found in their research that in complex tasks γ- tACS stimulation first improves accuracy in working memory rather than reflecting time (Santarnecchi et al., 2013). Hoy et al.'s study is similar to the above. When Hoy used γ- tACS to stimulate the F3 region, she found that it did not improve overall working memory performance, nor did it have a significant effect on improving task response time, but it could incresae higher working memory loads (Hoy et al., 2015).

At present, the relationship between the stimulation effect of gamma band tACS and the state of the brain when stimulated is not clear. Most scholars believe that the effect of tACS stimulation on working memory is influenced by the state of the brain at that time. When the brain is in a state of high cognitive load, the stimulation effect of gamma band is significant, but ceiling effect is prone to occur (Hoy et al., 2015; Roux et al., 2012). High working memory demands are typically associated with high-level frontal parietal connections, and synchronous tACS stimulation can enhance the entire brain network, thereby improving working memory levels (Violante et al., 2017). Some scholars also believe that the effect of tACS on working memory is correlated with the level of cognitive task participation (Polanía et al., 2012), and the deeper the level of cognitive participation, the greater the effect of tACS. Some scholars also believe that the effect of tACS stimulation is more likely to manifest only in individuals with poor working memory performance (Tseng et al., 2018).

In summary, this study indicates that the frequency of the impact of tACS on working memory is mainly concentrated in theta and gamma within the frequency band, and the stimulation area is mainly in the frontal and parietal lobes. When working memory performance is poor, the intervention effect of θ-tACS is better. When working memory is under high cognitive load, the intervention effect of γ-tACS is better, and the main improvement is the accuracy of working memory (Table 1).

**Table 1:** The effect of transcranial alternating current stimulation on working memory.

**References**	**Research object**	**Electrode position**	**Frequency (Hz)**	**Time (min)**	**Intensity (mA)**	**Electrode size**	**Cognition task**	**Effect of action**
Jaušovec and Jaušovec (2014)	36 healthy adults with an average age of 20 ± 4.25 years old	Left parietal cortex (P3)+supraorbital frontal cortex (F3); Right parietal cortex (P4)+supraorbital region	ITF	15	1–2.25 (pp)	(5^*^5) cm^2^	N-back task memory task	Significantly improved working memory performance
Violante et al. (2017)	24 healthy adults, 27.38 ± 4.56 years old	Frontal/parietal region (F4/P4), T8 F4, and P4, T8	6	20	1 (pp)	(5^*^5) cm^2^	2-back/1-back and selective reactions	Improving speech working memory performance
Wolinski et al. (2018)	Group 1 has an average age of 28.3 ± 7.6, Group 2 has an average age of 22.8 ± 5.2, and each group has 16 people	Parietal cortex (P4)+vertex (Cz)	4, 7	12	1.24 ± 0.3 mA	(5^*^5) cm^2^	Working memory task	4 Hz increases the working memory capacity, while 7 Hz reduces the shared working memory capacity.
Zeng et al. (2022)	36 healthy adults with an average age of 23.67 ± 1.97	FP1-AP7, FP2-AF8	4, 8, sham	20	2 (pp)	(4.5^*^5.5) cm^2^	N-back task memory task	8 Hz can improve performance in verbal n-back tasks
Pahor and Jaušovec (2018)	72 healthy female students with an average age of 20.38 ± 1.48	Group 1:P3-P4, Group 2:F3-P3, Group 3:F4-P4, Group 4:F3-F4	Group 1: θ4.94, γ31.81; Group 2: θ4.89, γ33.22 Group 2: θ5.08, γ32.60 Group 4: θ5.28	15	2 (pp)	(7^*^5) cm^2^	N-back task memory task	θ-tACS stimulation in the posterior parietal lobe enhances working memory
Zhang et al. (2022)	20 healthy young participants with an average age of 22.45 ± 2.52	F4, P4	6	15	2(pp)	(5^*^5) cm^2^	N-back task memory task	6 Hz stimulation has no significant effect on low-load working memory

ITF represents an individual θ Frequency; Pp represents the peak-to-peak value of the current; Sham represents false stimulation.

### 3.2 Impact of transcranial alternating current on learning ability

This study found that tACS can regulate neural activity through oscillating currents and improve the performance of motor learning. The synchronous oscillation activity at frequencies alpha (8–12 Hz) and beta (13–30 Hz) is believed to promote neuronal plasticity, thereby improving motor learning ability.

Pollok et al. found that tACS stimulation in the alpha frequency band (10 Hz) has a positive effect on learning motor sequences, and only when stimulating the parietal lobe can its effect be exerted. This is similar to the previous research results of Antal, in which Antal et al. found that tACS stimulation at 10 Hz has a significant promoting effect on implicit motor sequence learning (Pollok et al., 2015; Antal et al., 2008). Further research has found that tACS stimulation in the beta band (20 Hz) has a positive effect on learning stability and is less sensitive to interference. This is similar to Antal et al.'s previous research, in which Antal found that tACS stimulation at 10 Hz has a significant promoting effect on implicit motor sequence learning (Pollok et al., 2015; Antal et al., 2008).

Miyaguchi et al. (2018) found that tACS stimulation in the gamma band (70 Hz) can also improve the retention ability of motor learning by stimulating the M1 and cerebellar cortical areas, and the effect lasts for up to 24 h. This may be because tACS stimulation in the gamma band strengthens the neural network between M1 and the cerebellar hemisphere, and the neural network between M1 and the cerebellum is involved in monitoring motor errors and correcting motor planning, which is crucial for motor learning. Moreover, during motor preparation and execution, the gamma band activity in the M1 region increases, promoting information transmission in the sensory motor integration process (Pollok et al., 2015; Antal et al., 2008). During exercise tasks, the activity of the beta and gamma bands at M1 mutually inhibits each other. The application of γ-tACS on M1 may increase the activity of the gamma band while suppressing the activity of the beta band. This may be one of the reasons why gamma tACS (70 Hz) stimulation only improves the ability to maintain motor learning, which is consistent with the cross theory proposed by Pahor and Jaušovec (2014). Zhang et al. conducted experiments from the perspective of consolidating motor skills, and the results also support this conclusion. Research has found that within the same time window, compared to low-frequency (20 Hz) stimuli, high-frequency (70 Hz) stimuli have a greater effect and longer duration. Zhang et al. believes that when 70 Hz tACS stimulated the M1 region, **γ-**tACS increased brain gamma band activity and inhibited beta band activity, causing neurons related to motor learning and memory to be repeatedly stimulated by tACS, thereby promoting enhanced motor learning (Zhang et al., 2022). Actually, tACS stimulation in the alpha, beta, and gamma bands can all enhance motor learning ability. Pollok colleagues found that tACS stimulation at 10, 20, and 35 Hz can all improve motor learning ability. Compared to 10 and 20 Hz, the effect of 35 Hz is weaker. The reason why Pollok's research results differ from other scholars may be that Pollok places the stimulation position of tACS in the cortical layer of the first interfemoral muscle, while the experimental paradigm of motor learning ability is achieved through finger tapping on the keyboard. tACS intervention can provide strong stimulation to the cortical layer, and improve finger flexibility, and therefore all three stimulation frequencies can improve motor learning ability (Pollok et al., 2015). However, 20 Hz has the best effect on improving motor learning ability and has good anti-interference ability. Unlike the above, in Antal et al.'s study, 15 and 30 Hz tACS stimulation did not affect learning motor sequences, which may be due to the endogenous oscillatory state of the subject's brain, resulting in differences in effects between different studies (Antal et al., 2008). John Nguyen et al.'s study showed that when HD-tACS was applied to the medial frontal cortex (MFC) and lateral prefrontal cortex (lPFC) of subjects with open eyes, their learning ability was significantly improved. Further research has found that when subjects close their eyes, applying the same stimulation in the same position does not improve their learning ability (Nguyen et al., 2018). The reason may be that eye-opening behavior affects the neural network, leading to synchronization of the active theta band in the frontal lobe, thereby promoting functional connectivity between MFC and lPFC. This change is crucial for completing learning tasks, further proving that the endogenous oscillatory state of the subject's brain is an important factor affecting the effectiveness of tACS intervention.

In summary, the evidence provided in this study indicates that the brain regions that enhance motor learning ability are mostly selected as M1 or frontal cortex. alpha, beta, gamma band tACS stimulation has a positive effect on motor learning; θ-tACS on the frontal cortex improves rule learning ability, but at the same time interferes with the application of learning rules; β-tACS has the most stable effect on motor learning; The effect of γ-tACS on motor learning has a longer time effect, however, the endogenous oscillation state of the subject's brain can also affect the intervention effect (Wischnewski et al., 2016; Table 2).

**Table 2:** The effect of transcranial alternating current stimulation on learning ability.

**References**	**Research object**	**Electrode position**	**Frequency (Hz)**	**Time (min)**	**Intensity (mA)**	**Electrode size**	**Cognition task**	**Effect of action**
Nguyen et al. (2018)	30 healthy individuals with an average age of 24 years old	MFC and Right LPFC	6	20	1(pp)	NG	Time estimation task	Improved learning ability
Pollok et al. (2015)	13 healthy individuals with an average age of 22.08 years	Left M1 and above the right eye socket	10, 20, 35 and shame	12 min 12 s	1(pp)	(5^*^7) cm^2^	SRTT	10 and 20 Hz tACS promote learning of motion sequences
Miyaguchi et al. (2018)	30 healthy individuals with an average age of 21 ± 0.36 years	Right M1, left cerebellar cortex area	70	(60^*^8)S, 2 min apart each time	1 (pp)	(5^*^5) cm^2^	Visual motion control tasks	The error rate of sports learning is significantly reduced
Wischnewski et al. (2016)	50 healthy individuals with an average age of 24.1 ± 7.80 years old	At the frontal cortex between F3 and Fc5, and between F4 and Fc6	6	11	1 (pp)	(5^*^7) cm^2^	Reverse learning tasks	Reverse learning speeds up
Zhang et al. (2022)	14 healthy individuals with an average age of 22.53 ± 0 56 years old	Left M1 and above the right eye socket	20, 70 and sham	11	2 (pp)	(5^*^5) cm^2^	SRTT	Both 20 and 70 Hz can improve motor skills and sequence response skills, and the effect of 70 Hz is more significant
Antal et al. (2008)	16 healthy individuals with an average age of 22.4 ± 4.15 years old	Left M1 and above the right eye socket	1, 10, 15, 30, 45, and sham	5	0.4(pp)	(4^*^5) cm^2^ and (5^*^10) cm^2^	SRTT	10 Hz tACS can shorten reaction time and improve implicit motion learning
Minpeng et al. (2019)	60 healthy individuals with an average age of 20–25 years old	Left and right primary motor cortex, ipsilateral supraorbital region	20	15	Sensory stimulus intensity	(5^*^5) cm^2^	SRTT	Improved motor learning ability and shortened reaction time

FDI represents the first interosseous dorsal muscle; SRTT represents the sequence reaction time task; The intensity of sensory stimulation starts at 20 μ Step A increases the amplitude of the current, and when the subject has a slight pricking sensation or visual hallucinations on the scalp, increase it by 20 μ The step size of A decreases until the stimulus current disappears in the subject's sensation.

### 3.3 Impact of transcranial alternating current on decision-making ability

This study found that tACS stimulation of the frontal lobe brain area can improve sport decision-making ability. Sela et al. used balloons to simulate risk tasks, stimulating the left and right prefrontal cortex separately. One group received stimulation in the right prefrontal cortex (rPFC), and tACS stimulation was performed 5 min before the start of the task until BART was completed. The stimulation frequency of tACS was 6.5 Hz, and the final results showed that tACS was stimulated in the left prefrontal cortex (lPFC) theta frequency band neural oscillations can improve the ability of motor decision-making and prompt subjects to take action (Feurra et al., 2012). The research results of Dantas et al. are different from the above. When Dantas applies θ-tACS stimulation to the left prefrontal lobe, their risky decision-making behavior decreases, the reason may be that the experimental paradigms of the two are different. Dantas's experimental paradigm is a gambling task, which avoids the impulses of loss and disgust. Marco discovered the correspondence between the frontal striatum and hippocampus through his study of EEG-fMRI, demonstrating that tACS stimulation may increase decision-making motivation by indirectly affecting brain regions of the reward system (Dantas et al., 2021). More importantly, the ventral striatum is a key subcortical area for risk decision-making, and its activation indicates the making of risk decisions, making it more likely to be activated as rewards increase (Niv et al., 2012). The study by Yaple et al. supports this viewpoint that when Yaple uses different frequencies of tACS to stimulate the frontal area, a 20 Hz left frontal lobe stimulation can significantly increase the motivation for risk decision-making. This may be because a 20 Hz stimulation may increase cortical excitability in the left frontal lobe region by driving the frontal striatal network, which can enhance the motivation for risk decision-making (Yaple et al., 2017).

The decision-making process involves multiple brain regions, including the frontal lobe, parietal lobe, insula, caudate nucleus, amygdala, and anterior cingulate gyrus. The frontal lobe plays a significant role in decision-making (Gold and Shadlen, 2007). Rao et al. demonstrated a link between the PFC and the decision to voluntarily accept greater risk, suggesting that the PFC in the prefrontal cortex is more closely related to accepting greater risk and that the PFC regulates the active volitional control of risk recipients by executing control parts (Rao et al., 2008). Further research has found that in decision-making contexts, right PFC activation is considered withdrawal decision-making behavior, while left PFC activation promotes decision-making behavior (Davidson, 2014). Student's research results differ from the above, as he found that compared to the left prefrontal cortex, the right prefrontal cortex (rPFC) has a higher theta Power (4–8 Hz), higher frontal theta band asymmetry, and more adventurous behavior in decision-making tasks (Studer et al., 2013). The reason may be that there are differences in the resting state of the individual's frontal lobe, which can affect one of the key factors in decision-making behavior. Therefore, when using tACS for intervention, the first step should be to conduct EEG testing to exclude individual differences in the resting state of frontal lobe asymmetry (Slovic, 1966).

In summary, this study indicates that the stimulation of the left frontal lobe by tACS is an important area for improving decision-making ability, and the theta frequency band is an important frequency band for stimulating frontal lobe activation. However, the asymmetry of the frontal lobe and differences in resting state are also factors that affect decision-making behavior (Table 3).

**Table 3:** The impact of transcranial alternating current stimulation on motor decision-making.

**References**	**Research object**	**Electrode position**	**Frequency (Hz)**	**Time (min)**	**Intensity (mA)**	**Electrode size**	**Cognition task**	**Effect of action**
Sela et al. (2012)	27 healthy individuals with an average age of 23.89 ± 2 45 years old	Group 1: DLPFC(F3), (CP5), Group 2: DLPFC(F4), (CP6)	6.5	15	0.5 (pp)	(5 cm × 5) cm^2^	BART	Stimulation of the right frontal lobe reduces motivation for risk decision-making behavior
Yaple et al. (2017)	Group 1: 17 healthy individuals with an average age of 20.52 ± 2.52 years old; Group 2: 17 healthy individuals with an average age of 21.17 ± 2.78 years old	Group 1: F3, ipsilateral deltoid muscle; Group 2: F4, ipsilateral deltoid muscle	5 10 20 40	40	0.5 (pp)	(5 cm × 7) cm^2^	Risk Decision Tasks for Voluntary Conversion Tasks	20 Hz excitation of the left prefrontal cortex increases motivation for risk decision-making
Wischnewski et al. (2016)	18 healthy individuals with an average age of 21.9 ± 2.3 years old	Left and right prefrontal cortex; AF3 and AF4 outer 2cm, Fc1 and Fc2 outer 1 cm	5	30	0.5 (pp)	(3 cm × 5) cm^2^	Modified version of sequential gambling task	Frontal lobe θ-tACS can increase the perception of uncertainty in adventure missions
Dantas et al. (2021)	31 healthy adults with an average age of 23.8 ± 3.45	The large electrode is on the left DLPFC, and the small electrode is on F3	Shame, 6.5, 40	30	0.5 (pp)	Electrode with a diameter of 2.1 cm and a circular ring with an outer diameter of 11 cm and an inner diameter of 9 cm	Cambridge Gambling Mission	6.5 Hz reduces motivation for adventurous behavior

BART represents balloon simulation risk task.

## 4 Impact of cognitive function on sports performance

### 4.1 Impact of working memory on sports performance

Working memory is the ability to temporarily store, acquire, and process information in the brain in order to achieve higher-level cognitive functions. It is the foundation of learning and decision-making abilities, and all sports skills and performance develop based on working memory (Ren et al., 2013). Therefore, working memory is crucial for movement. The frontal and parietal lobes are the main functional areas of working memory tasks (Palva et al., 2010). The executive function is related to the frontal lobe, while the storage of working memory is related to the parietal lobe (Champod and Petrides, 2010). When individuals perform complex tasks, the storage capacity of working memory is crucial for task performance, such as basketball and football; for easier tasks, control is dominant, such as high jump and long jump (Jaušovec et al., 2014).

In competitive sports, phenomena related to working memory, such as choking and stereotype threat, can affect sports performance (Hardy et al., 1996). The phenomenon of choking consumes cognitive resources, reducing working memory capacity and impairing athletic performance. Stereotypes are similar to choking, which can cause individuals to refocus on well-practiced sensorimotor skills, interfering with their automatic execution and reducing working memory capacity, thus decreasing sports performance (Beilock et al., 2006). Athletes with lower working memory capacity are more likely to experience decreased accuracy, poor decision-making, or choking under high-pressure conditions (Hardy et al., 1996). Working memory itself may fluctuate due to situational stress, which can affect decision quality and, consequently, performance outcomes. Furley and Memmert found that elite basketball players are better able to concentrate on decision-making tasks while ignoring distractions, scoring higher on working memory capacity tests (Furley and Memmert, 2010). Another way working memory affects performance is through attentional control (Eysenck, 1998). Compared to players with lower working memory capacity, those with higher levels are better at focusing attention and making sound decisions in everyday life (Broadbent et al., 1982).

Furleyand and colleagues found through their research on ice hockey players that working memory capacity can predict the degree to which players adjust their decision-making behavior based on real-life scenarios. Research has shown that ice hockey players with high working memory capacity are able to autonomously adjust inappropriate attack plans and usually do not blindly follow predetermined tactical guidance (Furley and Memmert, 2012). Bisagno et al. found through regression research that working memory can serve as a predictor of volleyball performance, with higher levels of working memory indicating better volleyball performance (Bisagno and Morra, 2018). Wood et al.'s research suggests that individuals with smaller working memory capacity are more likely to experience anxiety and attentional impairment in stressful environments, thereby affecting athlete performance on the field (Wood et al., 2016). The results of this study indicate that working memory capacity can not only predict an individual's ability to control attention well but also predict athletes who may fail under high stress. The impact of working memory on sports performance is also reflected in the training stage. Coaches often provide specific instructions during practice or competition, and guide athletes to enter a prepared state of exercise, thereby helping athletes reduce the complexity of decision-making. This approach improves athlete performance by directing their attention in a targeted manner.

Compared to projects with higher levels of automation, tactical decision-making sports rely more on working memory. Mayers et al. found through cross-sectional research that there is a positive correlation between working memory and the performance of football players, and a higher level of working memory can enhance the performance of football players (Mayers et al., 2011). Some scholars have also found in their research that after cognitive-motor dual-task training (including working memory), basketball control performance is better than the group without cognitive-motor dual-task training because cognitive training stimulates the cognitive function necessary for fast and accurate basketball dribbling (Bisagno and Morra, 2018). But it is also related to the state of cognitive load. When the cognitive load is low, individuals have a greater ability to mobilize and control cognitive resources, improve working memory efficiency, and create possibilities for athletes to perform exceptionally well (Botvinick et al., 2001). On the contrary, it will weaken the efficiency of working memory and lead to abnormal motor performance (Baumeister, 1984). Therefore, enhancing working memory ability with tACS is beneficial for improving sports performance.

### 4.2 Impact of learning ability on sports performance

Learning ability is an important component of cognitive ability and the main way of acquiring motor skills. Learning ability helps to consolidate motor memory, reduce the attention required during exercise, make the movement more automated, improve the economy of movement, and in sports, it manifests as quickly acquiring motor skills and shortening the adaptation cycle (Fresnoza et al., 2020).

Nitsche et al. (2003) argue that the acquisition and early consolidation stages of motor skills require the involvement of motor learning. Using tDCS to stimulate the M1 brain area can significantly improve the performance level of motor learning, and promote the acquisition and maintenance of motor skills. Hillman et al.'s research also supports this point, believing that learning ability plays a role in skill acquisition. Higher learning ability can encourage athletes to be better at acquiring sports skills through observation, imitation, and analysis, and can quickly reach the autonomous stage of mastering sports skills, reduce cognitive load during exercise, and thus improve sports performance (Hillman et al., 2008). Kidgell et al. (2013) demonstrated through experiments that stimulating the M1 region with tACS (unilateral and bilateral) can improve learning ability. When completing Purdue pegboard test, motor performance significantly improves, with sustained effects reaching up to 60 min. Scholars such as Zhu used cathode tDCS to stimulate the left DLPFC, which also improved performance in sports learning and golf putting practice (Zhu et al., 2015). The reason may be that the enhancement of learning ability helps cultivate athletes' ability to correct sports movements, reduce errors in sports events, and improve the accuracy and economy of movements (McCullagh and Weiss, 2001). Faubert (2013) believe that athletes with fast learning abilities in unpredictable and complex dynamic scenes have better competitive performance. Moreover, in the study, it was found that professional athletes with stronger learning abilities completed better tracking tasks when completing multi-objective tracking tasks. On the other hand, some scholars believe that improving learning ability can help athletes better manage emotions and stress, improve self-efficacy, increase their confidence in completing actions, effectively reduce negative emotional interference, and ultimately improve sports performance (Starek and McCullagh, 1999). The above studies all indicate that the enhancement of learning ability is beneficial for athletes to quickly master sports skills and reduce movement errors in sports, which has a positive effect on improving sports performance.

There is a correlation between working memory and new skill learning. Recent research has begun to explore the role of working memory in motor learning, finding that working memory capacity can predict the learning outcomes of categorization tasks and the ability to solve mathematical problems (Beilock and Carr, 2005). Working memory plays a role in both visuomotor adaptation and motor sequence learning, particularly in the early stages of learning. Greater working memory capacity leads to stronger learning abilities, which in turn significantly enhance sports performance (Anguera et al., 2010). Therefore, tACS that boosts learning ability can improve sports performance.

### 4.3 Impact of decision-making ability on sports performance

Decision-making is a cognitive process of making choices between two or more options. Sports decision-making is an advanced stage of cognitive processing for athletes, which is a more comprehensive ability compared to working memory and learning ability. Therefore, decision-making requires the participation of more brain regions, which is very common in sports such as football, basketball, volleyball, etc. that require cooperation from multiple people (Hwang et al., 2022).

Short decision-making time is a characteristic of the sports field, especially in competitive sports. For complex and open sports, the level of sports decision-making directly affects the performance of athletes in terms of technical skills and athletic performance (Fu, 2004). On the sports field, athletes instantly integrate their own and opponent's situational information, perform high-speed and efficient processing, and quickly make judgments. Athletes with higher decision-making abilities can quickly and accurately make judgments and decisions on current tasks, avoiding choking effects and maintaining or even improving sports performance (Wang, 2013). On the contrary, incorrect sports decisions may choose the wrong tactics or techniques, which will directly lead to a decline in sports performance and even affect the score of the game. In high-level competitive events, the outcome of the competition does not depend on the factors of athletic skills, but on the choice of skills and tactics by the athletes. Elite athletes exhibit both accurate and reasonable decision-making performance because they have a reasonable cognitive structure toward sports scenes, and they can effectively allocate attention resources based on their cognitive advantages (Humphreys and Revelle, 1984).

Some scholars believe that the differences in sports performance among elite athletes are caused by differences in information selection and decision-making (Yan and Zheng, 2008). Some scholars even believe that there is a positive correlation between sports performance and sports decision-making. Athletes with higher sports decision-making abilities are faster in cognitive processing, and the higher the speed of sports decision-making, the higher the level of sports; When the decision-making ability of sports decreases, the accuracy of sports decision-making decreases, and the decision-making time becomes longer, which will reduce sports performance. Therefore, the level of sports decision-making ability can to some extent distinguish the level of athlete sports (Gilovich, 1984). In football matches, the level of athletic decision-making determines the upper limit of an athlete's athletic level at the same technical level (Xuanpeng, 2024). Sports decision-making ability enables athletes to make the best choices in evaluating potential risks and benefits, reducing the occurrence of sports errors due to blind decision-making. Huijgen et al. (2015) found in football that good sports decision-making is related to the athlete's accurate evaluation of the ball's trajectory and ability to catch the ball, as well as the athlete's ability to choose the best passing time and place teammates in the best scoring position. It is important for the performance of football players, and when decision-making ability decreases, it can affect the athlete's decision to make accurate shooting targets. The decision-making speed in complex tactics can be enhanced with training, and the decision-making speed is also an indicator of the level of sports skills. Li (2019) found in their research that sports decision-making training can effectively improve the decision-making speed and accuracy of basketball players during the passing process, thereby enhancing their performance on the field. When sleep deprivation leads to a decrease in the speed of sports decision-making, basketball players' performance also decreases. Amann et al. found in their research that exercise decision-making is crucial for endurance performance, as it determines whether to continue with endurance exercise (Amann and Secher, 2010). The above research indicates that sports decision-making not only affects individual sports performance but also affects group exercise performance. Therefore, enhancing exercise decision-making ability is beneficial for improving exercise performance.

The capacity of working memory is closely related to decision-making, and working memory is the guarantee of information processing in the decision-making process (Kane et al., 2007). Athletes with low working memory capacity are more likely to make decision errors in stressful situations, and improving their working memory capacity can improve sports decision-making (Chi et al., 2014). Furley and Memmert observed the relationship between working memory capacity and the anti-interference ability of ice hockey players in complex decision-making tasks by setting up interference scenarios (Furley and Memmert, 2012). The results showed that the high working memory capacity group had a higher probability of correct decision-making than the low working memory capacity group. The reason is that sufficient working memory capacity is the fundamental guarantee for timely updating competition information and making correct decisions in complex sports decision-making scenarios. Therefore, it can be seen that working memory capacity can play a positive role in sports decision-making. However, some research results are different from the above. For example, athletes are prone to the phenomenon of “choking” at critical moments in a competition, which is mainly influenced by the capacity of working memory. Athletes with higher working memory are often more likely to perform poorly in competitions. A possible explanation for this phenomenon is that athletes with higher working memory tend to use the working memory system when solving problems during the competition. Once the stress and anxiety of the competition interfere with the normal operation of the working memory system, it will cause a sudden decline in the athlete's sports decision-making ability, ultimately leading to a decline in sports performance (Chen and Liu, 2009; Wang, 2003). The above studies all indicate that the enhancement of sports decision-making can improve sports performance.

With the increasing recognition and support of coaches and athletes for the idea that “competition requires cognitive ability,” how to use tACS to enhance cognition has become a common concern for researchers, coaches, and athletes.

## 5 Development of intervention plans for tACS

In practical applications, it has been found that the intervention effect of tACS on cognition exhibits high variability, among which stimulus frequency, stimulus location, stimulus intensity, stimulus time, and athlete's state are important factors affecting the intervention effect (Klink et al., 2020). However, the intervention plan for tACS has not been standardized yet, and there are differences in intervention plans among different cognitive functions. Therefore, clarifying the constituent elements of the tACS intervention plan and its impact on cognitive function can help develop a refined tACS intervention plan, thereby improving the safety factor and application effect of tACS.

### 5.1 Stimulation frequency

Unlike other TES, tACS regulates brain oscillations through a unique current frequency, and brain oscillations of different frequencies are closely related to cognitive function. Therefore, the current frequency of tACS can significantly affect cognitive function (Morillon et al., 2019; Table 4).

**Table 4:** The effect of different frequencies of transcranial alternating current stimulation on cognitive function.

**Frequency band**	**References**	**Frequency (Hz)**	**Cognitive tasks**	**Cognitive function**	**Intervention effect**
θ(4–7)	Wolinski et al. (2018)	4	Retrospective working memory	Working memory	Improve the working memory of the subjects
	Wischnewski et al. (2016)	5	A modified version of the sequential gambling task	Decision-making ability	Improved perception of uncertainty
	Violante et al. (2017)	6	Visual-spatial working memory task	Working memory	Improved working memory performance
	Wischnewski et al. (2016)	6	Reverse learning tasks	Learning ability	Reverse learning speeds up
	Dantas et al. (2021)	6.5	Cambridge Gambling Mission	Decision-making ability	Reduced motivation for risk decision-making behavior
α(8–13)	Borghini et al. (2018)	10	Retrospective working memory	Working memory	Improving the working memory of participants
β(14–30)	Yaple et al. (2017)	20	Risk decision-making for voluntary task-switching	Decision-making ability	Increased motivation for making risk decisions
	Zhang et al. (2022)	20	SRTT	Learning ability	Shortened reaction time
	Pollok et al. (2015)	20	SRTT	Learning ability	Can improve motor skills and sequence response skills
	Minpeng et al. (2019)	25	SRTT	Learning ability	Reduced learning response time for motion sequences
γ(30–80)	Pollok et al. (2015)	35	SRTT	Learning ability	The promotion effect on learning motion sequences is not significant
	Borghini et al. (2018)	40	Change detection task	Working memory	Improved working memory ability
	Zhang et al. (2022)	70	SRTT	Learning ability	Improved learning ability
	Miyaguchi et al. (2018)	70	Visual motion control	Learning ability	Significant reduction in task error rate

The cognitive function affected by different stimulation frequencies varies, and there are five commonly used tACS stimulation frequencies, among which delta frequency range is 0–4 Hz; theta frequency range is 4–7 Hz; alpha frequency range is 8–13 Hz; beta frequency range is 13–30 Hz; gamma frequency range is 30–80 Hz (Klink et al., 2020). Research has found that (1) tACS stimulation of the different frequency can affect different cognitive functions, theta frequency stimulation is mainly related to working memory, the position of the parietal lobe theta Oscillation can improve the performance of working memory (Tseng et al., 2018); alpha frequency stimuli are mainly associated with executive function, visual attention, and memory processes (Kim et al., 2017; Taylor and Thut, 2012; Mierau et al., 2017); beta frequency stimulation is related to attention, working memory, and executive control (Engel and Fries, 2010); gamma frequency stimulation is related to the processing of input information, working memory, and situational memory (Fries, 2015; Pina et al., 2018; Nyhus and Curran, 2010). Analyzing the above studies, it was found that most tACS frequencies can affect working memory, possibly due to the wide band of working memory, and multiple frequencies of tACS can cause oscillations in the band of working memory. (2) Further research has found that different frequencies of tACS can also affect the same cognitive function, for example, some scholars have proposed beta stimulation can promote both motor learning and executive function (Schmidt et al., 2019), alpha stimulation can also inhibit executive function; When verifying the impact of α- tACS and θ-tACS on motion decision-making ability, Soutschek found no significant difference between the two (Soutschek et al., 2022). (3) Same frequency band will also have different effects on the same cognitive function. Wolinski found in his research that even if they all belong to the same category theta frequency of the band can also have a different effect on cognitive function. 4 Hz can deepen working memory, while 7 Hz cannot. (4) Stimulating effect of tACS is influenced by individual status. Due to the neural oscillation effect of input, the endogenous state of the subject's brain also greatly affects the intervention effect of tACS (Reato et al., 2013). For example, Axmacher and his colleagues found that as the workload of working memory increases, the theta frequency of the brain decreases, while there is no significant effect on the gamma frequency. When using tACS in the theta frequency band for stimulation, it does not always improve an individual's working memory ability (Axmacher et al., 2010). The θ-γ cross-frequency coupling theory posits that slower theta frequencies integrate more gamma cycles within each theta cycle, thereby increasing memory capacity (Lisman and Idiart, 1995). Conversely, faster theta frequencies incorporate fewer nested gamma cycles, leading to reduced memory capacity. Therefore, understanding individual differences is crucial for optimizing the effectiveness of tACS, which explains the variability in outcomes when the same stimulation is applied by different researchers (Sauseng et al., 2019). Fröhlich (2015) also suggest that tACS influences ongoing brain oscillations by altering their frequency, with the impact largely dependent on the brain's endogenous state and the EEG frequency of different functional regions.

Ali et al. (2013) believe that brain oscillatory activity is a periodic dynamic system that has an optimal response frequency. When the frequency of external stimuli reaches or approaches the resonance frequency of the brain network, the regulatory effect on neurons is strongest. Therefore, many scholars are committed to finding personalized tACS frequencies based on the endogenous oscillation frequency of the subject to increase the regulatory effect on cognitive function. Reinhart et al. confirmed the personalized internal frequency of each elderly subject by pre-collecting and analyzing task state EEG signals and then used this frequency as the stimulation frequency of high-precision tACS to regulate the working memory ability of the elderly. The results showed that the performance of working memory tasks significantly improved after stimulation and had better regulatory effects compared to fixed-frequency stimuli (Reinhart and Nguyen, 2019).

### 5.2 Phase

Among the factors that affect the effectiveness of tACS interventions, phase is often overlooked, but there is currently limited research on the phase of tACS. Due to the phase energy determining the relative positions of the peaks of endogenous and exogenous oscillations, the intervention effect of tACS is closely related to the endogenous oscillations in the brain, which can greatly affect the effectiveness of tACS.

In phase (phase difference of 0°) tACS stimulation can improve cognitive function, while out of phase (phase difference of 90 and 180°) tACS stimulation can reduce cognitive function. When the current waveform of tACS is sinusoidal AC, its phase can determine the intervention effect of tACS (Ishii et al., 1999). Placing the electrodes of tACS in different brain regions and setting their parameters can regulate the neural oscillatory activity between two brain regions, thereby altering information exchange between brain regions (Zaehle et al., 2010). For tACS stimulation in both brain regions, different phases of alternating current will produce different effects. Polanía et al. (2012) applied in-phase tACS (phase difference 0°), out-of-phase tACS (phase difference 180°), and false stimuli to the frontal and parietal regions of the subjects to investigate the effects of different phase tACS stimuli on executive function. The results showed that compared to false stimuli, in-phase stimuli significantly increased the response time of participants in task execution, while antiphase stimuli reduced task performance. This indicates that changes in the phase of tACS can affect the level of cognitive task completion. The study by Polania et al. also confirms this point, that in-phase theta frequency band tACS can reduce the reaction time in visual memory matching tasks, while the opposite reduces performance and increases reaction time (Polanía et al., 2012).

The synchronous phase stimulation of the frontal and parietal lobes can promote the improvement of working memory ability (Violante et al., 2017). Polania R's research found that the left prefrontal cortex and parietal cortex are in the same phase theta Stimulation can improve visual working memory, while the opposite is true theta Stimulation can reduce the performance of working memory (Polanía et al., 2012). Violante et al. further investigated the neural mechanisms underlying the impact of in-phase tACS on verbal working memory when stimulating the frontal and parietal lobes (Violante et al., 2017). The results indicate that the same phase frontal lobe θ-tACS stimulation can enhance the behavioral performance of working memory; The fMRI results showed that the same phase tACS stimulation in the frontal and parietal lobes can regulate brain activity and functional connectivity, and it was found that this regulatory effect is related to phase and the cognitive state of the subjects. That is, when the subjects perform high cognitive load tasks, the same phase tACS enhances the activation and functional connectivity of the frontal and parietal lobe brain regions, enhancing cognitive function. Some scholars also believe that the intervention effects of different tACS phases are related to the cognitive level of the subjects. Tseng found in his study of the impact of tACS on visual working memory that the same phase θ-tACS induces improvement in visual working memory performance, but only in low-level individuals, while high-level individuals may experience mild visual working memory damage. In another experiment, the reverse phase θ-tACS is not helpful for low-level individuals, but significantly impairs the visual working memory capacity of high-level individuals (Tseng et al., 2018). The reason may be that when cognitive function is at a high level, it often exhibits more complex neural signals. When using reverse tACS stimulation, may damage the phase relationship between endogenous and exogenous factors in high-performance individuals, reducing the brain's ability to process information (Costa et al., 2002).

Tseng et al. (2018) believe that the phase of brain oscillations reflects the encoding and retrieval status of the brain network, and the relative phase of oscillations increases the success rate of encoding and retrieval in the memory process. Therefore, when using the same phase Tacs to stimulate the frontal and parietal lobes, can improve the subject's working memory ability. Similarly, Sauseng et al.'s study found that when participants performed visual-spatial working memory tasks, there was a difference between the frontal and parietal lobes theta frequency band exhibits phase synchronization characteristics, indicating that phase synchronization within the Fronto Parietal Network (FPN) can improve the maintenance time of information in the working memory process (Tseng et al., 2018). Daume also found phase synchronization between the frontal and temporal lobes when studying them. Daume et al. (2017) used Magneto encephalo graphy to investigate brain activity during the retention phase of a delayed-match task. The results revealed phase synchronization between the left inferior temporal cortex and the prefrontal cortex in the lower frequency bands (θ/α bands) during the retention period. Additionally, they observed increased phase-amplitude coupling between the phases of theta and alpha bands and the amplitude of the beta band in the left inferior temporal cortex.

Phase differences are not solely related to exogenous frequencies but are also influenced by the endogenous frequencies of individual brain regions. When exogenous and endogenous frequencies align, the likelihood of phase synchronization increases. Reinhart et al. analyzed task-state EEG signals to determine each older participant's individualized internal frequency. This frequency was then used as the stimulation frequency for high-definition transcranial alternating current stimulation (HD-tACS) to modulate working memory performance in older adults. The results indicated that personalized HD-tACS could restore theta band phase synchronization between the frontal and temporal lobes during the retention period. Additionally, there was a significant increase in θ-γ phase-amplitude coupling (PAC) in the temporal region, leading to improved performance on working memory tasks (Reinhart and Nguyen, 2019).

In summary, in-phase tACS stimulation is beneficial for enhancing cognitive function, while anti-phase tACS stimulation can decrease cognitive function. When in-phase tACS is applied to the frontal and parietal lobes, the intervention has a greater impact on executive function and working memory. The effectiveness of in-phase tACS intervention is also related to the individual's cognitive level; the lower the cognitive level, the better the in-phase tACS intervention works.

### 5.3 Brain stimulation areas

The brain stimulation area is the area directly in contact with tACS stimulation. The cognitive functions represented by different brain regions in the brain are both overlapping and different. This study found that different brain regions have their advantages in cognitive functions. Therefore, exploring the stimulation of tACS in different brain regions is of great significance for targeted improvement of cognitive function. At present, in the intervention plan of tACS, the main brain regions stimulated are the frontal and parietal lobes. When stimulating the frontal lobe, the effects are diverse and can improve cognitive functions such as motor decision-making, working memory, motor learning, and attention; when stimulating the parietal lobe, the effect is relatively single, mainly having a positive effect on working memory (Table 5).

**Table 5:** The effect of transcranial alternating current stimulation on cognitive function in different brain regions.

**References**	**Brain region Effect of action**	**Electrode position**	**Cognitive function**	**Intervention effect**
Zhang et al. (2022)	Left motor cortex	Left M1 and above the right eye socket	Sports learning	Shortened reaction time
Nguyen et al. (2018)	Frontal cortex	MFC and Right LPFC	Learning ability	Improve learning ability
Sela et al. (2012)	Frontal cortex	DLPFC, F3	Sports decision-making	Reduce motivation for sports decision-making
Yaple et al. (2017)	Left frontal lobe	F3, Ipsilateral deltoid muscle	Sports decision-making	Increase motivation for sports decision-making
Yaple et al. (2017)	Right frontal lobe	F4, Ipsilateral deltoid muscle	Sports decision-making	Reduce motivation for sports decision-making
Wischnewski et al. (2016)	Prefrontal cortex	AF3 and AF4 outer 2cm, Fc1 and Fc2 outer 1cm	Sports decision-making	Increase the ability to perceive uncertainty
Jaušovec and Jaušovec (2014)	Left frontal lobe	F3; Right supraorbital forehead	Working memory	Improve working memory performance
Violante et al. (2017)	Frontal, parietal, and temporal lobes	(F4/P4), T8	Working memory	Improving speech working memory performance
	Left frontal and parietal lobes	DLPFC(F3), (CP5)	Sports decision-making	Reduce motivation for sports decision-making
Borghini et al. (2018)	Parietal cortex	P3, P4	Working memory	Improve working memory performance
Tseng et al. (2018)	Parietal cortex	(P3/P4), Left cheek	Visual working memory	Improve working memory performance
Bender et al. (2019)	Parietal cortex	(P4) + (Cz)	Working memory	Improve working memory performance

Research has found that both the frontal and parietal lobes have a positive effect on working memory, with the parietal lobe playing a central role in working memory. Jaušove et al. found that tACS stimulation on the left (P3) or right parietal lobe (P4) had a positive effect on working memory, but no such positive effect was observed on left frontal lobe (F3) stimulation (Jaušovec et al., 2014). Violante also proved this point, finding that in demanding working memory tasks, the right frontal-parietal network associated with task activation has a direct connection with brain synchronization (Violante et al., 2017). Further research has found a high correlation between the right frontal-parietal brain area and memory function, and the parietal lobe plays a central role in influencing memory function (Curtis and D'Esposito, 2004). Vossen applied 6 Hz tACS stimulation to the left frontal lobe (F3) and left parietal lobe (P3) cortex, respectively, and found that only when tACS was applied to the left parietal cortex did the visual working memory storage capacity improve. This supports the central role of the parietal lobe brain region in working memory storage capacity, and this finding has been confirmed in multiple neuroimaging studies (Vossen et al., 2015; Champod and Petrides, 2010). The impact of tACS electrode stimulation on cognitive function varies in different regions of the brain. Stimulating the frontal and parietal regions at F3-P3 may regulate the frontal striatal network related to motor decision-making, or the frontal parietal network related to voluntary executive control (Rao et al., 2008). When stimulated in F3-F4, it can modulate the frontal and deep medial structures (Bai et al., 2014), while the F3-P3 electrode may modulate the frontal and parietal structures, indicating that stimulating the left frontal lobe or inhibiting the right frontal lobe increases decision-making ability (Orr and Banich, 2014). Stimulation of both the frontal and parietal lobes can enhance working memory ability, but the parietal lobe is the core area that enhances working memory. Parietal lobe θ-tACS stimulation improves the accuracy of working memory, while frontal lobe stimulation θ-tACS stimulation shortens the working memory response time, indicating that even if the same cognitive function is affected by different brain regions, there are differences in the changes in cognitive function.

Numerous studies by scholars have shown that the frontal lobe is related to motor decision-making and plays an important role in voluntary risk decision-making. For example, Rao et al. (2008) demonstrated a link between the prefrontal cortex and voluntary acceptance of greater risk, suggesting that the prefrontal cortex regulates the active willpower control of risk-takers by controlling executive components. The ventral striatum is located behind the frontal lobe, and activation in this area indicates the making of risk decisions. As decision rewards increase, the probability of activation also increases. Therefore, stimulating the frontal lobe may enhance decision motivation because tACS stimulation affects the deep ventral striatum position (Rao et al., 2008; Niv et al., 2012). Moreover, due to the asymmetry of the brain, even when electrical stimulation is performed in different brain regions of the same lobe, the stimulation effect varies. Sela et al. θ-tACS is applied to the left or right dorsolateral prefrontal cortex, and participants perform decision-making tasks that require risk-taking. Sela used tACS with a frequency of 6.5 Hz and an intensity of 1 mA to provide stimulation for 15 min during the task. A group of subjects received tACS stimulation in the left prefrontal cortex, while a group of subjects received tACS stimulation in the right prefrontal cortex. The results showed that only the stimulation in the left hemisphere had a significant impact on motor decision-making. Compared with the stimulation in the right hemisphere and false stimuli, participants with left stimulation had higher motor decision-making motivation (Sela et al., 2012). This discovery has also been confirmed by other scholars. When Dantas stimulated the left frontal lobe in the experiment, the motivation for adventurous motor decision-making decreased; when stimulating the right frontal lobe, the motivation for adventurous exercise decision-making increased. This may indicate a potential functional correlation between asymmetry in the left and right frontal lobes and risky decision-making (Dantas et al., 2021; Badre et al., 2012).

Some scholars also believe that the relationship between stimulating brain regions and cognitive function cannot be clearly defined. The reason may be that the maximum value of tACS stimulation is not located at the stimulation site. Bikson et al. modeled the current in the brain and found that when stimulating the primary motor cortex, the maximum current value does not lie below the electrode, but spreads to the frontal cortex (Bikson et al., 2010); Moreover, the brain is a complex network structure, and there is currently a lack of clear understanding on whether the stimulation of a specific brain area alone causes changes in the activity of other brain areas, thereby enhancing cognitive function. Therefore, it cannot be determined whether the change in cognitive function is a unique effect of tACS stimulation on a specific brain area, or whether similar effects can be observed by stimulating other cortical areas. Scholar Okada believes that the stimulation of brain regions by tACS is not a simple change in the stimulated area, but rather a strengthening of the connections between different brain regions. However, most experiments have not monitored changes in oscillatory activity or excitability during the stimulation process, and there is a lack of direct evidence to prove that excitability changes in the target area affect cognitive function. Therefore, clarifying the relationship between stimulated brain regions and cognitive function is still under exploration (Okada et al., 2004).

In summary, the reason why the frontal and parietal lobes are ideal targets for tACS stimulation may lie in two aspects: (1) because their anatomical location is relatively shallow and easy to approach; (2) Neuroimaging studies have shown that the frontal lobe can affect a wide range of cognitive functions, including working memory, attention, learning, creative thinking, and social functioning. Therefore, most current studies support the frontal lobe as the main stimulus area affecting cognitive function (Duncan and Owen, 2000).

### 5.4 Stimulation dose

The size of the stimulation dose is related to the stimulation intensity, stimulation time, and electrode size, and is an important factor affecting the stimulation effect. A single stimulation plan not only cannot improve the application effect of tACS but may even cause irritating injury to the subjects (Peterchev et al., 2012). Based on previous research, most current studies have found that the stimulation parameters are as follows: the peak current intensity is between 0.5 and 2 mA, the stimulation time is between 6 and 40 min, and the electrode size is between 9 and 35 cm^2^. The most commonly used parameters are: the peak current is 1 mA, the stimulation time is 20 min, and the electrode size is 35 cm^2^.

With the deepening of research, scholars have found that different stimulation doses may have different effects on cognitive function. More accurate stimulation doses are conducive to the application effect of tACS in cognitive function. Currently, current intensities with peak values of 0.5–2 mA are commonly used in clinical practice (Table 6). The current intensity in this area can not only have a good intervention effect but also avoid skin burns caused by excessive stimulation intensity. The changes in cortical excitation depend on changes in tACS intensity. For example, a low stimulation intensity of 0.4 mA can lead to an increase in cortical excitation threshold, which in turn reduces excitability (Moliadze et al., 2012); Scholar Vöröslakos believes that a high current should be used. Low current intensity cannot overcome the consumption of scalp/skull shunting, and residual current cannot affect cortical excitation. Therefore, a higher current intensity may be needed to fully overcome skin/skull shunting, and it is recommended that the stimulation intensity of the current be above 2 mA (Vöröslakos et al., 2018). Moreover, when the stimulation is sufficiently strong, new neural oscillations can be triggered by the current intensity, making the intervention more effective. However, excessive current can also stimulate the skin and cause damage, so increasing the current intensity to enhance the effect while ensuring safety is a future research direction (Liu et al., 2018). Stimulation time is also an important factor affecting intervention effectiveness (Stagg et al., 2018). The intervention time of tACS is usually divided into two types: one is synchronous with cognitive task time, and the other is a fixed time, usually 20 or 30 min, with a single time usually not exceeding 40 min. The reason is that long-term stimulation of tACS can disrupt the stable state of synapses, leading to a decrease in intervention effectiveness (Batsikadze et al., 2013).

**Table 6:** Common different stimulation doses of tACS.

**References**	**Cognitive function**	**Current intensity (mA)**	**Stimulation duration (min)**	**Electrode size (cm^2^)**
Jaušovec and Jaušovec (2014)	Working memory	1–2.25 (pp)	15	All 5 ^*^ 5
Tseng et al. (2018)	Working memory	1.6 (pp)	20–24	(4^*^4), (5^*^7)
Violante et al. (2017)	Working memory	1 (pp)	20	All 5 × 5
Bender et al. (2019)	Working memory	2 (pp)	Synchronize with tasks (60 task experiments)	19.6, 4.9
Borghini et al. (2018)	Working memory	1.5 (pp)	20	All 5 × 7
Wolinski et al. (2018)	Working memory	1.24 ± 0.3 mA	12	All 5 × 5
Pollok et al. (2015)	Sports learning	1 (pp)	12 min 12 s	All 5 × 7
Zhang et al. (2022)	Sports learning	1 (pp)	11	All 5 × 7
Minpeng et al. (2019)	Sports decision-making	0.5 (pp)	15	All 5 × 5
Wischnewski et al. (2016)	Sports decision-making	0.5 (pp)	30	All 3 × 5

At present, there are no strict regulations on the size of electrodes. However, research suggests that under the premise of a certain current, the size of the electrode will affect the current density, and current density is a key factor affecting individual tolerance. Moreover, current density is also influenced by current intensity, and there is currently a lack of research on current density. Therefore, most studies on stimulus dose focus on stimulus intensity and duration. At present, some studies suggest that traditional large electrodes (5 ^*^7 cm^2^) can cause current dissipation and affect other brain regions, making it difficult to confirm the detailed relationship between regulatory effects and cognitive function. Therefore, to clarify this relationship of changes, more small-area stimulation electrodes are used. Dmochowski found in his research that when multiple small electrodes are used, the stimulation target of tACS is more focused, and the stimulation effect can be significantly enhanced (Dmochowski et al., 2011). Therefore, many scholars have also called for the use of small electrodes with multiple stimulation sites for tACS electrodes. However, currently, most studies still use large-area stimulation electrodes, possibly because the mechanism of action of tACS is to induce endogenous oscillations in the brain through alternating current, rather than relying on stable electric fields to stimulate the brain. However, currently most studies still use large-area stimulation electrodes, possibly because the effectiveness of tACS is related to the frequency of tACS and independent of electrode size. The second reason may be that large-area electrodes are more convenient to operate than small-area electrodes.

In summary, tACS stimulation should have different doses for specific cognitive functions. The basic principle is to first select the target area for stimulation based on the regulated cognitive function, then select the size of the electrode and determine the intensity of the stimulation to avoid electrical stimulation injury; finally, select the stimulation time based on the cognitive task (Table 6).

### 5.5 Other factors affecting the effectiveness of tACS intervention

Based on formulating intervention plans for tACS, the brain state, timing of application, and target audience will also affect the intervention effect of tACS. Research has found that: (1) the intervention effect of tACS is related to individual cognitive needs, and the higher the cognitive needs, the better the effect of tACS intervention on improving cognitive function (Violante et al., 2017). The reason may be that when an individual's cognitive function is worse, there is a greater demand for cognition, making it difficult to achieve the “ceiling” effect. Therefore, tACS has a greater effect on improving cognitive function (Kardos et al., 2014; Moliadze et al., 2019). (2) The effect of tACS on cognitive function is also influenced by eye state. Nguyen J et al. found that 6 Hz tACS enhances learning ability when subjects are in an eyes-open state. However, when subjects are in an eyes-closed state, the enhancement in learning ability is not significant. This may be due to the eyes-open state enhancing executive processes, promoting neuroplastic changes in theta functional connectivity between the MFC and lPFC, thus improving learning ability (Nguyen et al., 2018). (3) The application timing of tACS can be divided into before the task, during the task, and after the task; in practical applications, it is mostly before and during the task. (4) The application of tACS in different populations also has differences in effectiveness. The effect of tACS on patients with cognitive impairment is higher than that on healthy individuals, and its effect is more significant in the elderly population (Qi et al., 2023).

## 6 Ethical risks of using transcranial alternating current

“Putting people first” is the origin of competitive sports and the foundation for the healthy development of competitive sports. Therefore, for ethical considerations, it is crucial to explore whether the use of tACS will violate the regulations of the World Anti-Doping Agency (WADA) regarding its use. According to the requirements of the WADA code (The World Anti-Doping Agency, 2020), if a substance or method meets any two of the three standards, it will be considered for inclusion in the International Standard Prohibited List of the WADA (hereinafter referred to as the Prohibited List). According to the “Prohibited List,” prohibited substances and methods are classified as prohibited within the competition, prohibited on all occasions, and prohibited for special projects (Imperatori et al., 2018). Current research indicates that tACS can improve athletic performance by enhancing cognitive abilities, and no actual or potential harm to the health of athletes has been found when using tACS. Therefore, whether tACS will be banned by the World Anti-Doping Agency largely depends on whether its use violates the spirit of sports.

After research, it was found that tACS may be inconsistent with the advocated sportsmanship in the following three aspects.

- May have increased inequality. tACS, which can improve cognitive function, may also increase competitive inequality among athletes. Due to the different costs of obtaining tACS, athletes from different countries and regions may have differences in their use of tACS. This may result in some athletes being able to easily use tACS while others are unable to use it, leading to athletes who can use tACS devices gaining an advantage in competition. The application of tACS may also result in training inequality. Some scholars have found in their research that tACS can enhance cognitive function, while others have not found that tACS cannot enhance cognitive function (Pollok et al., 2015; Wischnewski et al., 2016). Therefore, the effect of tACS on exercise performance has both positive and negative aspects. The reasons for this include the intervention plan of tACS, the state of athletes, etc. Therefore, the statement that tACS improves exercise performance is not entirely consistent, which is not a problem in scientific research, but it is very important in the recognition of the World Anti-Doping Regulations. If athletes can improve their athletic performance without working hard during training, it will increase the inequality among athletes during training.
- There is a risk of policy loopholes. At present, there is no known method that can reliably detect whether an individual has recently undergone optimization with tACS stimulation, making neural stimulants almost undetectable, making it impossible to confirm whether an individual has used tACS to improve cognitive function before the competition (Park, 2017). When tACS emerged as a new technology, there was an imbalance between existing anti-doping policies and emerging technologies, and existing policies could not explain well whether tACS had violated the World Anti Excitement Regulations (Rodenberg and Hampton, 2013). For example, due to the lack of advanced testing methods to identify athletes who may abuse this technology, genetic stimulants were not included in their banned technology list until 2009 (Fore, 2010). However, some scholars have used monitoring BDNF to determine whether individuals have used tDCS intervention, which provides direction for monitoring the use of tACS. However, further empirical evidence is still lacking (Donati et al., 2021). Perhaps there will be methods for detecting nerve stimulants in the future, but they should be as cheap and easy to obtain as possible to ensure the people-oriented spirit of sports.
- Clarify what sports spirit is. There is no clear standard for evaluating sportsmanship in the World Anti-Doping Regulations, and there may be differences in the understanding of sportsmanship between athletes and the World Anti-Doping Agency. How to use tACS without violating the sportsmanship spirit of the World Anti-Doping Regulations? Imperatori once stated in his discussion of the application of tDCS that even small changes to tDCS can have a significant impact on sports outcomes for elite athletes. Therefore, it is recommended to only use tACS in training, while it should be prohibited in competitions. tACS is similar to tDCS and seems to be subject to similar constraints (Imperatori et al., 2018). However, there is indeed a lack of clear guidelines for the use of tACS (Pugh and Pugh, 2021). At present, scholar Qi and colleagues pointed out in their research that since tACS does not cause actual or potential harm to the health of athletes, nor does it violate the spirit of sport, it does not violate the two-thirds rule of the World Anti-Doping Regulations and is not currently classified as a stimulant (Pugh and Pugh, 2021). However, the spirit of sports is a moral standard related to the value and significance of sports, and there is no consensus on how to evaluate it (Loland and McNamee, 2019).

In summary, tACS does not violate the World Anti-Doping Code in current regulations. However, due to the complexity of tACS intervention programs and the significance of improving exercise performance, the World Anti-Doping Agency may not completely ban the use of tACS in the future but will take certain measures to limit its use, such as allowing the use of tACS to assist training only during the exercise training phase. Therefore, the World Anti-Doping Agency should organize scholars to research the use of tACS as soon as possible, develop a scientifically comprehensive tACS guidance manual, regulate the use of tACS, and minimize the damage to sportsmanship caused by the use of tACS.

## 7 Reflection and outlook

At present, there is a lack of research on the intervention effects of combining tACS with other treatment methods, and most studies focus on analyzing the individual intervention effects of tACS. There is also a lack of tracking reports on the effectiveness of tACS interventions, and monitoring the effectiveness of tACS interventions is often limited to the same day. Therefore, more research is needed to understand how the subsequent effects of tACS stimulation change over a longer period, to provide more accurate guidance for the application of tACS. In the past, the subjects were mostly healthy individuals or college students, lacking attention to professional athlete groups. The research results under experimental conditions lacked ecological validity. Therefore, more professional athletes should be selected as subjects, and tACS should be applied in actual competitive sports to explore the impact of tACS on athletes' cognitive function, and through what pathways it affects sports performance, thus enriching the application scenarios of tACS and provide technological assistance for athletes to achieve excellent competitive results.

At present, most studies examine the effects of tACS from a behavioral perspective, without evaluating the effects of tACS from the perspective of motor cortical excitability. Therefore, it is difficult to further explain the relationship between behavioral manifestations and the impact of tACS on the neocortex. Moreover, the level at which tACS works is not yet clear. To elucidate the causal relationship between cognitive function and tACS oscillatory activity, it is necessary to apply motor behavior, electrophysiology, and electroencephalography techniques to result in interpretation (Elyamany et al., 2021).

## 8 Conclusion

tACS intervention is an important means of improving cognitive function, which can enhance the working memory, learning ability, and decision-making ability of athletes and healthy individuals, and has a positive effect on improving sports performance.

The factors that determine the effectiveness of tACS intervention include stimulation frequency, stimulation phase, stimulation area, stimulation dose, etc. The stimulation area and frequency determine which cognitive function tACS affects, whereas the stimulation phase and dose determine the magnitude of the intervention effect. Moreover, before applying tACS, individual cognitive status, age level, and timing of application should be included as factors that affect the effectiveness of tACS intervention, to develop more scientific intervention plans.

Although there is no evidence to suggest significant safety issues with the use of tACS, there are still potential safety risks associated with the promotion and use of tACS among athletes. At present, there is no authoritative organization in China that provides clear operational guidelines for the application of tACS, and there is a lack of safe range values for tACS stimulation parameters. When used in the field of competitive sports, whether it will be recognized as a “nerve stimulant” or have its competition results canceled, authoritative institutions must clarify the usage scenarios of tACS and develop testing equipment for tACS to ensure that the use of tACS intervention can be known.

## Data Availability

The original contributions presented in the study are included in the article, further inquiries can be directed to the corresponding author.
